# Optimizing *de novo* genome assembly from PCR-amplified metagenomes

**DOI:** 10.7717/peerj.6902

**Published:** 2019-05-09

**Authors:** Simon Roux, Gareth Trubl, Danielle Goudeau, Nandita Nath, Estelle Couradeau, Nathan A. Ahlgren, Yuanchao Zhan, David Marsan, Feng Chen, Jed A. Fuhrman, Trent R. Northen, Matthew B. Sullivan, Virginia I. Rich, Rex R. Malmstrom, Emiley A. Eloe-Fadrosh

**Affiliations:** 1 Department of Energy Joint Genome Institute, Walnut Creek, CA, United States of America; 2 Department of Microbiology, Ohio State University, Columbus, OH, United States of America; 3 Environmental Genomics and Systems Biology, Lawrence Berkeley National Laboratory, Berkeley, CA, United States of America; 4 Department of Biology, Clark University, Worcester, MA, United States of America; 5 Institution of Marine and Environmental Technology, University of Maryland Center for Environmental Science, Cambridge, MD, United States of America; 6 Department of Biological Sciences, University of Southern California, Los Angeles, CA, United States of America; 7 Department of Civil, Environmental and Geodetic Engineering, Ohio State University, Columbus, OH, United States of America

**Keywords:** Metagenomics, Microbial ecology, Genome assembly, Viral metagenomics

## Abstract

**Background:**

Metagenomics has transformed our understanding of microbial diversity across ecosystems, with recent advances enabling *de novo* assembly of genomes from metagenomes. These metagenome-assembled genomes are critical to provide ecological, evolutionary, and metabolic context for all the microbes and viruses yet to be cultivated. Metagenomes can now be generated from nanogram to subnanogram amounts of DNA. However, these libraries require several rounds of PCR amplification before sequencing, and recent data suggest these typically yield smaller and more fragmented assemblies than regular metagenomes.

**Methods:**

Here we evaluate *de novo* assembly methods of 169 PCR-amplified metagenomes, including 25 for which an unamplified counterpart is available, to optimize specific assembly approaches for PCR-amplified libraries. We first evaluated coverage bias by mapping reads from PCR-amplified metagenomes onto reference contigs obtained from unamplified metagenomes of the same samples. Then, we compared different assembly pipelines in terms of assembly size (number of bp in contigs ≥ 10 kb) and error rates to evaluate which are the best suited for PCR-amplified metagenomes.

**Results:**

Read mapping analyses revealed that the depth of coverage within individual genomes is significantly more uneven in PCR-amplified datasets versus unamplified metagenomes, with regions of high depth of coverage enriched in short inserts. This enrichment scales with the number of PCR cycles performed, and is presumably due to preferential amplification of short inserts. Standard assembly pipelines are confounded by this type of coverage unevenness, so we evaluated other assembly options to mitigate these issues. We found that a pipeline combining read deduplication and an assembly algorithm originally designed to recover genomes from libraries generated after whole genome amplification (single-cell SPAdes) frequently improved assembly of contigs ≥10 kb by 10 to 100-fold for low input metagenomes.

**Conclusions:**

PCR-amplified metagenomes have enabled scientists to explore communities traditionally challenging to describe, including some with extremely low biomass or from which DNA is particularly difficult to extract. Here we show that a modified assembly pipeline can lead to an improved *de novo* genome assembly from PCR-amplified datasets, and enables a better genome recovery from low input metagenomes.

## Introduction

Microbes and their associated viruses dominate all ecosystems on Earth and drive major biogeochemical cycles (Falkowski, Fenchel & Delong, 2008; Suttle, 2007). The vast majority of this microbial and viral diversity has not yet been cultivated (Schloss et al., 2016; Lloyd et al., 2018), hence metagenomics, i.e., the sequencing of genomes directly from environmental samples, has emerged as a key method to explore these communities (Raes & Bork, 2008; Edwards & Rohwer, 2005). Briefly, DNA is extracted from an environmental sample, sometimes after selecting a subset of the community (e.g., the viruses), and sequenced, typically as short sequencing “reads”. These reads are assembled into larger contigs, interpreted as genome fragments, which provides the foundation to investigate functional, ecological, and evolutionary patterns of the largely uncultivated microbial and viral diversity (Schloissnig et al., 2013; Sunagawa et al., 2015; Tyson et al., 2004; Parks et al., 2017; Anantharaman, Breier & Dick, 2016; Burstein et al., 2016; Hug et al., 2016; Dutilh et al., 2014; Roux et al., 2016a; Spang et al., 2015).

Problematically, as metagenomics is applied to a broader set of samples, some yield very little DNA (e.g., a few nanograms), which poses a challenge for library construction (Rinke et al., 2016). Examples include low-biomass environments like ice cores or clean rooms (Knowlton et al., 2013; Weinmaier et al., 2015), tough-to-sample locations like hydrothermal vents (Anantharaman, Breier & Dick, 2016), and sampling procedures that target subsets of the community, e.g., virus particles or labeled metabolically active microbes (Duhaime et al., 2012; Hatzenpichler et al., 2016). Sequencing libraries from these types of samples require a DNA amplification step either before or after adapter ligation. In the former, extracted DNA is subjected to whole genome amplification (WGA), typically as Multiple Displacement Amplification (MDA)(Yokouchi et al., 2006) or Sequence-Independent, Single-Primer Amplification (SISPA)(Reyes & Kim, 1991). The resultant amplified product is then sufficient for a standard library preparation and sequencing. However, strong amplification biases make these approaches unsuitable for quantitative estimations of taxa or genes relative abundance (Marine et al., 2014; Bowers et al., 2015). Alternatively, tagmentation or adaptase protocols allow sub-nanogram DNA inputs for adapter ligation, and then use PCR (typically ≥ 9 cycles) to amplify the ligated DNA (Rinke et al., 2016; Roux et al., 2016b). In contrast to whole genome amplification, these protocols yield metagenomes (hereafter “PCR-amplified metagenomes”) for which read mapping enables a quantification of taxa and/or genes, and are thus the methods of choice for low-input metagenomes. (Rinke et al., 2016; Bowers et al., 2015).

While the impact of PCR amplification, sequencing library choice, and sequencing platforms on metagenome reads composition has been extensively studied (e.g., Rinke et al., 2016; Bowers et al., 2015; Duhaime & Sullivan, 2012; Solonenko et al., 2013), and specific assemblers have been developed for unamplified and MDA-amplified metagenomes (e.g., Nurk et al., 2017; Nurk et al., 2013), evaluation of *de novo* genome assembly from PCR-amplified metagenomes is needed. Here we compared different approaches for *de novo* assembly of PCR-amplified metagenomes generated with two library preparation kits commonly used on low input samples (Nextera XT and Accel-NGS 1S Plus). We show that preferential amplification of short inserts can lead to uneven genome coverage and sub-optimal assembly. We then highlight alternative sequence processing approaches that maximize *de novo* genome assembly for PCR-amplified libraries, which will enable scientists to extract as much information as possible from these datasets.

## Materials & Methods

### Origin of samples

Samples and libraries generated as part of 6 different projects were used in this study (Table S1). Most of these samples yielded a low amount of DNA, mainly because they targeted a specific community subset such as viruses, cyanobacteria, or metabolically active cells.

The data analyzed here included:

(i) A set of 20 samples from virus fractions along a natural permafrost thaw gradient (“Permafrost-associated viruses” in Table S1). These were generated using a protocol optimized for recovery of soil viruses (Trubl et al., 2016) with minor amendments. Briefly, viruses were resuspended from triplicate soil samples using a combination of chemical and physical dispersion, filtered through a 0.2 µm polyethersulfone membrane filter, and viral DNA was extracted using DNeasy PowerSoil DNA extraction kit (Qiagen, Hilden, Germany, product 12888). Extracted DNA was quantified using a Qubit-fluorometer (Invitrogen) following the manufacturer’s instructions.

(ii) A set of 14 samples from the viral fraction of Delaware Bay Estuary surface water (“Delaware Bay viruses”). These surface water viral metagenomes were collected during different seasons from the Delaware estuary and Chesapeake estuary using a Niskin bottle on board of the RV Hugh R Sharp. Details of environmental conditions can be found at http://dmoserv3.bco-dmo.org/jg/serv/BCO-DMO/Coast_Bact_Growth/newACT_cruises_rs.html0%7Bdir=dmoserv3.whoi.edu/jg/dir/BCO-DMO/Coast_Bact_Growth/,info=dmoserv3.bco-dmo.org/jg/info/BCO-DMO/%20Coast_Bact_Growth/new_ACT_cruises%7D. Viral communities were concentrated from 0.2 µm filtrates following the FeCl3 flocculation method (John et al., 2011). Briefly, 10 L of seawater was prefiltered through a 142 mm-diameter glass fiber filter GA-55 (∼0.6 µm-pore size, Cole-Parmer) and a 0.22 µm-pore-size Millipore polycarbonate membrane filter (142 mm, Millipore) to remove larger organisms and bacteria. One mL of 10g/L FeCl3 stock solution was added to the 10 L filtrate. After incubating with FeCl3 for 1 hr, the concentrated viral fraction was collected using a 0.8 µm-pore-size Millipore polycarbonate membrane filter (Millipore). The concentrated viruses were resuspended using a resuspension buffer, dialyzed to remove the resuspension buffer, and treated with DNase to remove free DNA. The viral DNA was extracted using the phenol-chloroform-isoamyl alcohol method. DNA concentrations were quantified using a NanoDrop 2000 spectrophotometer (Thermo Scientific, Walter, MA, USA) following the manufacturer’s instructions.

(iii) A set of 11 samples from the viral fraction of surface water at the San Pedro Ocean-time Series site (33°33′N, 118°24′W), off the coast of Los Angeles (“SPOT viruses”). Surface water was collected using a Niskin bottle rosette (5 m) or by bucket (0 m). Viral fraction (<0.22 µm) material was obtained using a peristaltic pump to prefilter seawater through a 0.22 µm Sterivex filter cartridge (EMD Millipore) then collection of 0.5 to 1 L of filtrate on a 25 mm 0.02 µm Whatman Anotop filter cartridge (GE Life Sciences). DNA from the Anotop cartridge was extracted using the protocol “Extracting nucleic acids from viruses on a filter” in ref. (Steward & Culley, 2010). DNA concentrations were determined using the Quant-iT™ PicoGreen™ dsDNA Assay Kit (Invitrogen) following the manufacturer’s instructions, with the fluorescence of samples and standards measured in a 96-well plates on a Stratagene MX-3000 quantitative PCR machine.

(iv) A set of 18 samples from North-American freshwater lakes (Lake Erie, Lake Michigan, and Lake Superior) from which cyanobacteria were selectively sorted using fluorescence activated single-cell sorting flow cytometry (“Freshwater cyanobacteria” in Table S1). For each sample, approximately 100,000 cells were sorted, and DNA was extracted using prepGEM (ZyGEM, Hamilton, New Zealand) on the cells pellet after 1 h centrifugation at 7,200 g and subsequent removal of supernatant.

(v) A set of 34 samples from Lake Mendota surface water, for which mini-metagenomes were generated by sorting individual gates using fluorescence activated single-cell sorting flow cytometry (“Mendota communities”). Briefly, subsets of the total microbial cells were defined based on a combination of fluorescence, forward scatter, and size scatter, to generate mini-metagenomes from 75,000 to 150,000 “similar” cells. DNA from these different cell pools was extracted using prepGEM (ZyGEM, Hamilton, New Zealand) on the cells pellet after 1 h centrifugation at 7,200g and subsequent removal of supernatant.

(vi) A set of 20 samples from desert soil microbial communities, from which mini-metagenomes were generated following incubation with a bio-orthogonal non-canonical amino acid (BONCAT, “Soil BONCAT”, Hatzenpichler et al., 2016; Couradeau et al., 2018). These samples were then sorted via fluorescence activated single-cell flow cytometry to separate active from inactive microbial cells. DNA was extracted from 100,000 sorted cells using prepGEM (ZyGEM, Hamilton, New Zealand) on the cells pellet after 1 h centrifugation at 7,200 g and subsequent removal of supernatant.

### Library construction and sequencing

Three library preparation methods were used here, including TruSeq DNA PCR-Free DNA Sample Preparation Kit (Illumina, San Diego, CA, USA), Nextera XT DNA Sample Preparation Kit (Illumina, San Diego, CA, USA), and Accel-NGS 1S Plus (Swift BioSciences, Ann Arbor, MI, USA). The only samples which contained enough DNA to create a TruSeq DNA PCR-Free library were some samples from the “Delaware Bay viruses” project, for which both Nextera XT and 1S Plus libraries were also created (Table S1). For the two other virus projects (“Permafrost-associated viruses” and “SPOT viruses”), both Nextera XT and 1S Plus libraries were created. Finally, Nextera XT libraries were created for all other projects (“Freshwater cyanobacteria”, “Mendota communities”, “Soil BONCAT”, Table S1). All libraries were prepared according to manufacturer’s instructions, and included as many PCR cycles as necessary to obtain 200 pM of DNA for sequencing, with a maximum of 20 cycles for viral metagenomes and 25 cycles for targeted microbial metagenomes. Finally, viral metagenomes were sequenced on either Illumina HiSeq-2500 or Illumina HiSeq-2000, and targeted microbial metagenomes with Illumina NextSeq HO, all with 2 × 151 reads (Table S1).

### Reads contamination filtering and trimming

For all libraries, BBDuk adapter trimming (bbduk.sh: https://sourceforge.net/projects/bbmap/ v35.79, parameters: ktrim=r, minlen=40, minlenfraction=0.6, mink=11, tbo, tpe, *k* = 23, hdist=1, hdist2=1, ftm=5) was used to remove known Illumina adapters. The reads were then processed using BBDuk quality filtering and trimming (parameters: maq=8, maxns=1, minlen=40, minlenfraction=0.6, *k* = 27, hdist=1, trimq=12, qtrim=rl). At this stage reads ends were trimmed where quality values were less than 12, and read pairs containing more than three ‘N’, or with quality scores (before trimming) averaging less than 3 over the read, or length under 51 bp after trimming, as well as reads matching Illumina artifact, spike-ins or phiX were discarded. Remaining reads were mapped to a masked version of human HG19 with BBMap (bbmap.sh v35.79, parameters: fast local minratio=0.84 maxindel=6 tipsearch=4 bw=18 bwr=0.18 usemodulo printunmappedcount idtag minhits=1), discarding all hits over 93% identity, per JGI standards procedure. Finally, for all Accel NGS 1S Plus libraries, the first 10 bases of forward and reverse reads were discarded to avoid contamination by the low complexity adaptase tail, per manufacturer’s instruction.

### Comparison of different assembly pipelines

The different assembly pipelines tested here included combinations of two types of read correction, two types of read selection or no read selection, and two types of assemblies (Table S2). The two methods used for read correction were chosen to represent either a “strict” or “relaxed” read correction. The “strict” correction used bfc (v. r181 (Li, 2015)) to remove reads with unique kmers (parameters: “-1 -s 10g -k 21″), followed by seqtk (v. 1.2-r101-dirty: https://github.com/lh3/seqtk) to remove reads for which paired sequences was removed by bfc (parameters: “dropse”). The “relaxed” read correction aimed at keeping as many reads as possible, and used tadpole.sh (v. 37.76: https://jgi.doe.gov/data-and-tools/bbtools/) to correct sequencing errors by leveraging kmer frequency along each read (parameters “mode=correct ecc=t prefilter=2″).

An additional read selection step was tested to check whether removing some of the reads associated with regions of high coverage could help *de novo* genome assembly. The two approaches evaluated here included read normalization with bbnorm.sh (v. 37.76: https://jgi.doe.gov/data-and-tools/bbtools/) in which the kmer-based read depth is leveraged to identify high-depth reads and normalized these to a defined depth (here 100x, parameters: “bits=32 min=2 target=100″), as well as a deduplication approach with clumpify.sh (v37.76: https://jgi.doe.gov/data-and-tools/bbtools/), in which identical reads are identified and only one copy retained (parameters: “dedupe subs=0 passes=2″). These parameters identify reads as duplicated only if they are an exact match (i.e., no substitution allowed). The ratio of duplicated reads was calculated by comparing the number of reads after deduplication to the number of input reads for each library (Table S1).

Finally, two different modes of the SPAdes assembler (v. 3.11 (Nurk et al., 2017; Nurk et al., 2013)) were tested to assess whether this could also influence assembly. Specifically, the two modes tested were metaSPAdes (option “–meta”) and single-cellSPAdes (option “–sc”). In both cases, SPAdes was run with the error correction step skipped (“–only-assembler”) and a fixed set of kmers (“-k 21,33,55,77,99,127”).

Assemblies were evaluated using a standard set of metrics computed with stats.sh from the bbtools suite (https://jgi.doe.gov/data-and-tools/bbtools/) and a custom perl script. These included cumulative size of all contigs, cumulative size of all contigs ≥ 10 kb, total number of contigs, minimal contig length among contigs making up to 50% of assembly size (N50), minimal contig length among contigs making up to 90% of assembly size (N90), and size of the largest contig (Table S2). Kolmogorov–Smirnov test (from the R package stats R Core Team, 2018) and Cohen’s effect size (as implemented in the R package effsize Torchiano, 2017) were used to compare distributions of cumulative size of all contigs ≥ 10 kb between different pipelines.

Assembly errors were estimated for the 25 libraries for which an unamplified library was available (Table S3) using QUAST (Mikheenko et al., 2018). All contigs ≥ 1 kb were included in this analysis, with contigs assembled from the corresponding unamplified library with a standard metagenome assembly pipeline (“strict” read correction, no read selection, and metaSPAdes assembly) used as a reference genome. QUAST was run with the “–fast” option enabled, all other parameters left to default. QUAST provides counts for three types of misassemblies: “relocation” in which two contiguous sections from a newly assembled contig map to the same reference sequence but non-contiguously, “inversion” in which two contiguous sections from a newly assembled contig map to the same reference sequence with one fragment being reversed, and “translocation” in which two contiguous sections from a newly assembled contig map to different contigs in the reference assembly. Because the assembly from unamplified libraries are not true reference genomes, i.e., each contig is not an independent chromosome, we ignored the misassemblies identified as “translocation”, as these could represent cases where both assemblies are correct and produced distinct but overlapping contigs, i.e., the new contig could genuinely match to the 5′and 3′edges of two distinct contigs from the reference assembly. Instead, the estimated rate of misassemblies was calculated for each assembly as the sum of the number of “relocations” and “inversions” provided by QUAST, divided by the total length of all contigs ≥ 1 kb.

### Coverage bias analysis

Quality-checked reads were mapped to reference assemblies to estimate contigs coverage and assess potential coverage biases along these contigs. For libraries for which an unamplified metagenome was available (i.e., the 11 samples from the “Delaware Bay viruses” project, Table S3), contigs from a standard metagenome assembly of the unamplified library were used as reference. For every other PCR-amplified library, contigs obtained through the “best” assembly pipeline, i.e., relaxed read correction with tadpole.sh (https://jgi.doe.gov/data-and-tools/bbtools/), read deduplication with clumpify.sh (https://jgi.doe.gov/data-and-tools/bbtools/), and assembly with SPAdes single-cell (error correction turned off, k-mers of 21, 33, 55, 77, 99, 127 Nurk et al., 2013) were used as reference. The mapping was computed using BBMap (bbmap.sh https://jgi.doe.gov/data-and-tools/bbtools/) with random assignment of ambiguously mapped reads (parameters: “mappedonly=t interleaved=t ambiguous=random”).

For contig coverage comparison to unamplified libraries (Fig. S1A), individual contig coverage was normalized by the library size (i.e., total number of bp in library). For estimation of coverage bias associated with high and low depth of coverage regions along individual contigs, bam files were parsed using a custom perl script to (i) identify unique mapping events, i.e., combinations of unique mapping start coordinate and insert size, and (ii) calculate for each unique mapping the number of different reads providing this exact mapping, the corresponding GC% of the insert, and the size of the insert. This was performed on all contigs ≥ 10 kb if these totaled ≥ 50 kb, or on all contigs ≥ 2 kb otherwise. For 3 libraries (BYXNC, BYXNG, and COHNO), no contigs ≥ 2 kb were generated, and the coverage bias was thus not estimated (Table S1).

To quantify the insert size bias, high and low depth regions were first defined for each contig as follows: inserts with a read depth ≥ 70% the maximum read depth of the contig were considered as high depth, while inserts with a read depth ≤ 30% of the contig maximum read depth were considered as low depth. For each library, the distribution of insert size for each of these two types of inserts was gathered, and these were compared using the non-parametric Kolmogorov–Smirnov test (from the R package stats; R Core Team, 2018). Cohen’s effect size (as implemented in the R package effsize; Torchiano, 2017) was also used to assess the magnitude of the difference between the means of the two distributions.

All graphical representations were generated with R (R Core Team, 2018) using the following packages: ggplot2 (Wickham, 2016), dplyr (Wickham et al., 2018), and RColorBrewer (Neuwirth, 2014).

### Partial proteins and genome binning evaluation

To assess the impact of the number of PCR cycles and assembly methods on contigs annotation, we evaluated the percentage of genes predicted as partial over the total number of predicted genes for each assembly. Genes were first predicted for all contigs ≥ 1 kb using Prodigal v. 2.6.3 with the “meta” option enabled (Hyatt et al., 2010). Predicted amino acid sequences were then compared to the UniProtKB/TrEMBL 2019_02 protein database (The Uniprot Consortium, 2019) using diamond v0.9.14 with default parameters for a random subsample of 20,000 predicted genes for each assembly. Genes predicted from the contigs were then affiliated to their best hit in UniProtKB/TrEMBL (cutoffs of 10^−5^ on e-value and 100 on score). Partial genes were identified by comparing the length of the predicted gene to the length of the reference best hit: after verifying that the distribution of this length ratio was centered around 1, we considered genes with a length <90% of the reference length as partial.

For microbial metagenomes (i.e., “Freshwater cyanobacteria”, “Mendota communities”, and “Soil BONCAT”), an estimation of the total number of distinct genomes assembled in contigs ≥ 1 kb was first obtained using Anvi’o v5.1.0 “anvi-run-hmms” and “anvi-display-contigs-stats” functions (Eren et al., 2015). These estimates are based on the detection of single-copy marker genes from bacteria (Campbell et al., 2011) and archaea (Rinke et al., 2013). Next, individual assemblies were binned using Metabat 2 v2.12.1 with default parameters (Kang et al., 2019), and the completeness and contamination of each genome bin was assessed using CheckM v1.0.12 with default parameters (Parks et al., 2015). Following recommendations from the MIMAG checklist (Bowers et al., 2017), genome bins with completeness >90% and contamination <5% were considered high-quality genome bins, ones with completeness ≥ 50% and contamination <10% were considered medium-quality bins, and ones with completeness <50% and contamination <10% were considered low-quality bins.

### Data availability

Reads for the different metagenomes are available on https://genome.jgi.doe.gov/portal/ and the SRA database (https://www.ncbi.nlm.nih.gov/sra), using the links listed in Table S1. Custom perl scripts used in this study are available at https://bitbucket.org/srouxjgi/scripts_pcrlibs_assembly_optimization/src/master/. Results from the different assembly pipelines are available for each library at http://portal.nersc.gov/dna/microbial/prokpubs/BenchmarksPCRMetagenomes/.

## Results & Discussion

Coverage biases and assembly pipelines were evaluated across 169 PCR-amplified metagenomes (Table S1). These included 87 viromes, i.e., virus-particle-enriched metagenomes, and 82 targeted microbial metagenomes, i.e., generated after flow cytometry cell sorting and representing only a small subset of the microbial community. Paired PCR-amplified metagenomes generated with the two common library preparation kits (Nextera XT and 1S Plus) were available and could be directly compared for 42 samples (Table S1). In addition, unamplified (TruSeq) libraries were available for 11 samples and used as a reference “standard metagenome” for these samples (Table S3).

### Insert length bias of PCR amplification leads to uneven coverage along genomes

Contrary to protocols including an amplification of the DNA pool prior to library construction (e.g., MDA, SISPA), the read composition of a PCR-amplified metagenome should accurately reflect the original community composition. This has been previously demonstrated (Rinke et al., 2016), and could be verified here by observing the coverage of reference contigs (obtained from unamplified metagenomes) in PCR-amplified metagenomes. Overall, nearly all contigs assembled from unamplified metagenomes were detected in PCR-amplified datasets (>90% of contigs with ≥ 5x average coverage depth, Table S3), and there was a strong correlation between unamplified and PCR-amplified coverage for shared contigs (average Pearson correlation *r*^2^ = 0.77 for Nextera XT and 1S Plus library methods, Table S3, Fig. S1).

**Figure 1:**
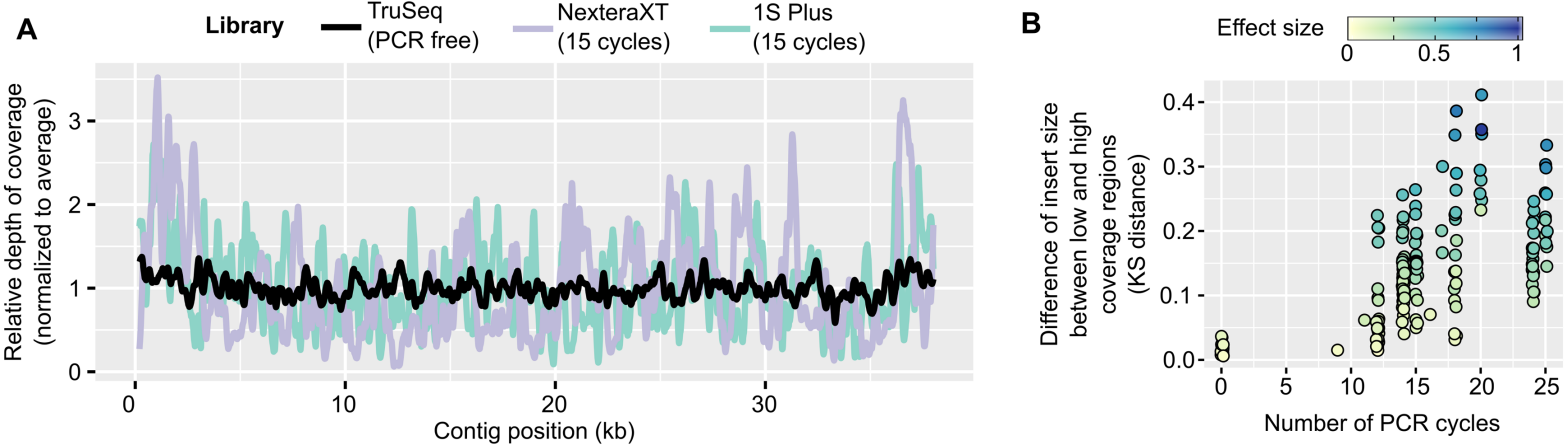
Coverage bias within individual contigs for unamplified and PCR-amplified libraries. (A) Example of coverage bias along a single contig from sample 1064195 (contig 1064195_contig_573). Reads from libraries ASXXB, BWNCO, and BWWYG (Table S2) were mapped to the same contig, and read depth along sliding windows of 100 bp is displayed for each library on the *y*-axis. Windows on the edges of the contig (within 200 bp of the 5′ or 3′ end) were excluded as read depth is not as reliable in these end regions. (B) Illustration of the insert size bias associated with high depth of coverage regions in PCR-amplified libraries. For each library, the number of PCR cycles performed for the library is indicated on the *x*-axis, while the Kolmogorov–Smirnov distance between the insert size distribution of low- versus high-depth regions is indicated on the *y*-axis. The magnitude of the difference between the means of the two distributions was also estimated using Cohen’s effect size (d) and is indicated by the dot color. For clarity, only libraries for which the mean insert size was lower in high depth regions are included in the plot, and the 22 libraries which showed the opposite trend are not plotted (Table S1). KS: Kolmogorov–Smirnov

In contrast, PCR-amplified metagenomes displayed a relatively high percentage of duplicated reads compared to unamplified datasets (∼25–85%, Fig. S1B), which contribute to an uneven depth of coverage along individual contigs (Fig. 1A). This unevenness can be measured through the coefficient of variation of coverage depth (standard deviation divided by average coverage, for each contig) which was relatively low for unamplified metagenomes (34% on average) but higher in all PCR-amplified libraries (58% average, 20–357% range, Table S1). Regions with high depth of coverage were not linked to any systematic GC bias but were enriched for short inserts (Fig. S2). As for the ratio of duplicated reads, the difference in insert size between high and low depth regions tended to increase with the number of PCR cycles performed (Fig. 1B). This suggests that some of the uneven coverage along genomes is due to over-amplification of short inserts, which make up a larger proportion of the read pool with each additional PCR cycle.

### *De novo* genome assembly can be improved using tailored read curation and assembly pipeline

Uneven coverage can hamper assembly because standard metagenome assembly pipelines expect a uniform coverage along each genome, and leverage this signal to solve repeats and ambiguities (Nurk et al., 2017). We thus looked at three data processing steps that could be customized for PCR-amplified libraries. First, standard metagenome assemblies typically use a strict read correction and remove reads with low depth which are potentially erroneous (Li, 2015). Even if these low-depth reads are correct, they represent low abundance sequences that would likely not assemble well anyway, and removing them reduces the time and resources (CPU and memory) required for the assembly. In the case of PCR-amplified libraries however, these low-depth reads might be important to retain, in order to correctly assemble even high-depth contigs (Fig. 1A). Second, read selection tools have been developed to either remove duplicated reads, or computationally normalize libraries, i.e., cap at a defined maximum depth. These tools have been primarily designed for MDA datasets, the majority of which deriving from single cell amplification, however these could be helpful as well for PCR-amplified metagenomes. Finally, some assemblers offer customized options for metagenomes and for single-cell (MDA) libraries, and we tested whether single-cell options might perform better on these PCR-amplified metagenomes.

**Figure 2:**
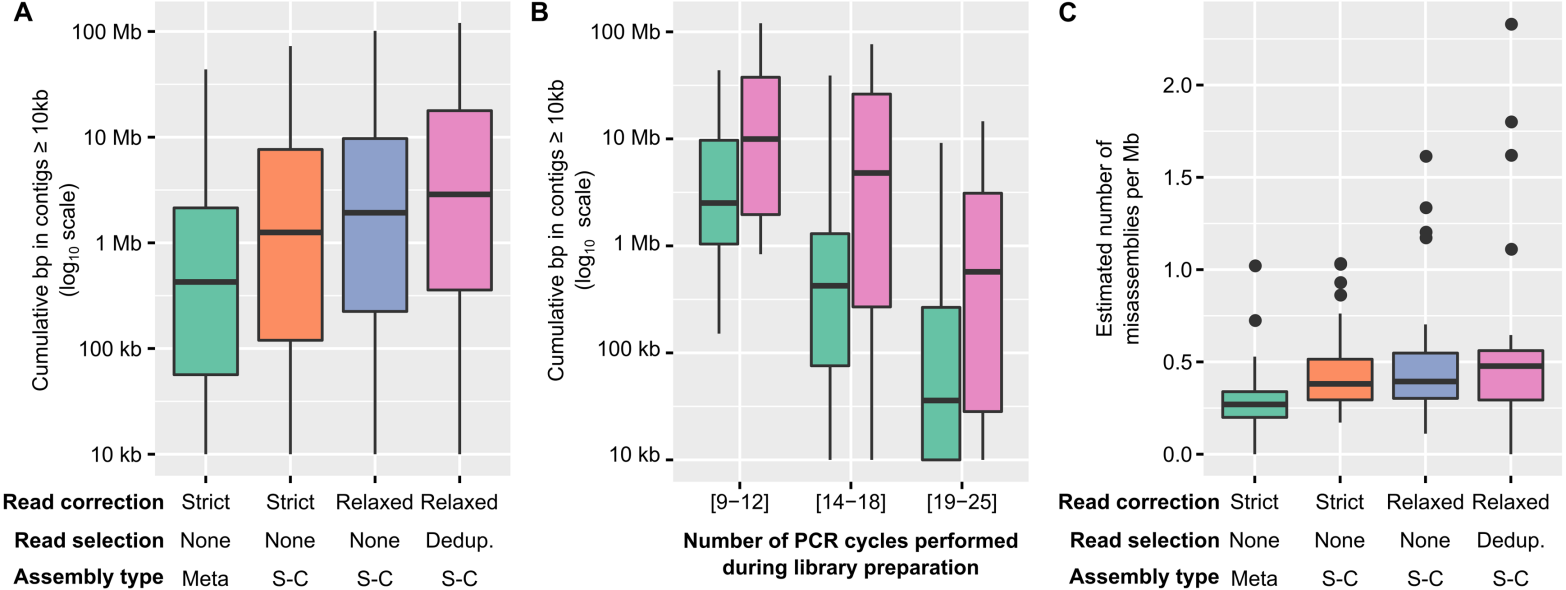
Optimized pipeline for assembly of PCR-amplified metagenomes. (A) Distribution of the cumulative size of long (≥10 kb) contigs (*y*-axis) obtained across all PCR-amplified libraries from different assembly pipelines (*x*-axis). Assembly pipelines are indicated along the *x*-axis (see Table S3). (B) Cumulative size of long (≥10 kb) contigs obtained with a standard (green) or optimized (purple) assembly pipeline for different ranges of library PCR amplifications (*x*-axis). Coloring of the assembly pipelines is identical as in panel A. (C) Estimated error rate (*y*-axis) from different assembly pipelines (*x*-axis) across all PCR-amplified libraries. These assembly errors were estimated for the 25 libraries for which an unamplified reference assembly was available (Table S2). Coloring of the assembly pipelines is identical as in panels A and B. Dedup.: Deduplication, Meta: metaSPAdes, SC: single-cell SPAdes.

Over the 12 combinations tested, a pipeline including “relaxed” read correction, read deduplication, and single-cell assembly parameters provided the largest assemblies, i.e., the ones with the largest cumulative length of contigs ≥ 10 kb, although the level of improvement varied (Fig. 2A, Table S2). While the cumulative length of contigs ≥ 1 kb only moderately increased compared to a standard assembly (median: 1.17x, Fig. S3A), the cumulative length of contigs ≥ 10 kb showed a much larger improvement (median: 3.6x, range: 0.95–3,806x, ks-test *p*-value: 1e^−07^, Cohen’s effect size: 0.66, Fig. S3B). Since large contigs tend to be more relevant for downstream applications, such as genome binning and annotation, systemically applying this “relaxed” read correction, read deduplication, and single-cell (i.e., “Corr_Dedup_SC”) assembly strategy on PCR-amplified metagenomes maximizes the information recovered from these datasets. Overall, when considering contigs ≥ 10 kb, the Corr_Dedup_SC strategy provided the largest assembly for 130 samples, and was within 80% of the largest assembly for another 17 samples (Fig. S3C), suggesting it would be a suitable default choice for any PCR-amplified metagenome.

To verify whether genome annotation and binning were indeed improved when using the Corr_Dedup_SC, we first evaluated the ratio of partial genes observed across different assemblies (Fig. S4A, Table S2). When compared to a standard assembly, the proposed Corr_Dedup_SC strategy provided a slight decrease (∼2–5%) in the percentage of partial genes (ks-test p-value: 1.8e^−08^, Cohen’s effect size: 0.57). This is most likely due to longer contigs leading to less genes being predicted on contig edges, and suggests that these Corr_Dedup_SC assemblies enabled improved annotation of the genome fragments assembled. Next, we estimated the number of distinct genomes and performed genome binning on the targeted microbial metagenomes (“Freshwater cyanobacteria”, “Mendota communities”, and “Soil BONCAT”) to compare genome recovery between the different assemblies (Figs. S4B and  S4C, Table S2). Both the total number of genomes in contigs ≥ 1 kb and the number of high- and medium-quality genome bins increased in the Corr_Dedup_SC compare to the standard assemblies, confirming that downstream genome annotation would be improved by applying this Corr_Dedup_SC assembly strategy (ks-test p-values: 3.4e^−05^ and 9.3e^−04^, Cohen’s effect size: 0.76 and 0.49, respectively).

The level of assembly improvement observed was in part linked to the number of PCR cycles performed for each metagenome (Fig. 2B, Table S2). Specifically, samples that required 9 to 12 PCR cycles typically assembled well with the standard metagenome pipeline, with 8Mb in contigs ≥ 10 kb on average, which was improved with the Corr_Dedup_SC assembly to an average of 26 Mb (Cohen’s effect size: 0.68). Samples that required 14 to 18 PCR cycles were improved further as standard assemblies yielded an average of 2Mb in contigs ≥ 10 kb per metagenome as compared to 15Mb from Corr_Dedup_SC assemblies (Cohen’s effect size: 0.9). Lastly, the assembly of samples that required 20 to 25 PCR cycles remained limited with either approach, though still slightly improved from 562 kb to 2 Mb in contigs ≥ 10 kb for the standard versus Corr_Dedup_SC approaches (Cohen’s effect size: 0.68). Similarly, the percentage of partial genes was higher in libraries with 20 to 25 PCR cycles compared to libraries with 9 to 12 PCR cycles, even in Corr_Dedup_SC assemblies (ks-test: 2.2e−16, Cohen’s effect size: 2.02, Fig. S4D).

Finally, we analyzed the samples for which both unamplified and PCR-amplified metagenomes were available to evaluate the error rate in assemblies obtained from the Corr_Dedup_SC strategy (Table S1). Specifically, we used QUAST (Mikheenko et al., 2018) to identify “relocation”, i.e., cases in which contiguous regions of a newly assembled contig are non-contiguous in the reference assembly, and “inversion”, i.e., cases in which the orientation of contiguous regions differs between the new assembly and reference contigs. This suggested that the Corr_Dedup_SC assembly strategy generated more erroneous contigs than a standard assembly pipeline (Cohen’s effect size: 0.7, Fig. 2C, Fig. S3D). For these metagenomes, the relative increase in error rate (median: 2x) remains much lower than the increase in number of long (≥10 kb) contigs (median: 24×, Table S2), so the Corr_Dedup_SC assembly strategy still seems to represent an acceptable trade-off between assembly size and assembly errors for most applications, yet this higher error rate must be considered when analyzing these datasets.

## Conclusions

The ability to prepare and sequence libraries from samples containing nanograms or less of DNA has been a tremendous advance for the fields of metagenomics and microbial ecology, and many biological insights have already been derived from these data. Here we highlight how a PCR amplification bias for shorter inserts can hamper standard *de novo* genome assembly for viral and microbial low-input metagenomes, and propose an Corr_Dedup_SC assembly strategy able to reduce its impact. This will aid scientists in maximizing genomic context from low input metagenomes, and should help improve understanding of challenging ecosystems and targeted subsets of microbial and viral communities.

##  Supplemental Information

10.7717/peerj.6902/supp-1 Figure S1 PCR-amplified metagenomes are quantitative but include a significant amount of duplicated reads (A) Comparison of depth of coverage between unamplified (TruSeq, *x*-axis) and PCR-amplified (Nextera XT or Accel-NGS 1S Plus, *y*-axis) libraries. The average depth of coverage was computed for each contig as the average read depth normalized by the total size of the library. The 1:1 equivalence is indicated with a black line, while a linear best fit is shown in blue. For clarity, only 1,000 contigs randomly selected from each sample are plotted. Contigs with no reads mapped in the PCR-amplified library were not included. To be able to directly compare the two plots, only samples for which both a Nextera XT and 1S Plus libraries were available are included (Table S1). The subpanels show the correlation coefficient (Pearson and Spearman) of a sample-by-sample correlation between depth of coverage in unamplified and PCR-amplified libraries, either for all contigs or only for contigs ≥ 10 kb with a depth of coverage ≥10 ×. (B) Percentage of duplicated reads (*y*-axis) as a function of the number of PCR cycles performed during library creation (*x*-axis). Underlying data are availabe in Table S1.

10.7717/peerj.6902/supp-2 Figure S2 Insert size and GC content distribution for all vs high-depth regions (A & B) Distribution of insert size for all regions (green) or only regions with high depth of coverage (orange) across PCR-amplified libraries. In panel A, all insert sizes were centered around 500 bp to enable a more direct comparison between libraries. Panel B shows the same data without this transformation (i.e. raw insert size). (C & D) Distribution of GC % for all regions (green) or only regions with high depth of coverage (orange). For panel C, each library GC% was centered around 50%, while panel D shows the same data without this transformation.

10.7717/peerj.6902/supp-3 Figure S3 Assembly size and estimated error rates for different assembly pipelines Comparison****of the output of different assembly pipelines applied to PCR-amplified libraries. Panels A & B show the cumulative length of all contigs (A) or contigs ≥ 10 kb (B) across assembly pipelines (*x*-axis). Panel C displays the cumulative length of contigs ≥ 10 kb relative to the largest value for each library, i.e. as a percentage of the “best” assembly for this library (“best” being defined as the largest cumulative length of contigs ≥ 10 kb). Panel D displays the distribution of estimated error rates across the different assembly pipelines, for the 25 libraries for which error rates could be estimated (Tables S2 & S3). Underlying data for individual assemblies are available in Table S2. Norm.: Normalization, Dedup.: Deduplication, Meta: metaSPAdes, SC: single-cell SPAdes.

10.7717/peerj.6902/supp-4 Figure S4 Impact of library PCR amplification and choice of assembly methods on contig annotation (A) Link between assembly methods choice and ratio of incomplete gene. The boxplot displays the estimated ratio of incomplete genes (see Methods) in each assembly relative to this ratio in the “standard” metagenome assembly (Strict-None-Meta). (B) Estimated number of bacterial and archaeal genomes in assemblies based on known single-copy marker genes. (C) Number of samples for which ≥ 1 high-quality or medium-quality genome bin(s) (bacterial or archaeal) was obtained. (D) Comparison of the ratio of incomplete genes for different number of PCR cycles, for contigs obtained using the “standard” assembly pipeline (“Strict-None-Meta, ”left panel) or the proposed “optimized” pipeline (“Relaxed-Dedup-SingleCell”, right panel). Underlying data for individual assemblies are available in Table S2. Norm.: Normalization, Dedup.: Deduplication, Meta: metaSPAdes, SC: single- cell SPAdes.

10.7717/peerj.6902/supp-5 Table S1 Description of samples and libraries analyzed The first tab lists information about individual samples including the list of all libraries generated for each sample, and the second tab includes information about each library.

10.7717/peerj.6902/supp-6 Table S2 Results from the different assembly pipelines tested The first tab lists the different steps and tools tested. The second tab includes the results of *de novo* genome assembly with the different pipelines for each PCR-amplified library, alongside the estimated ratio of incomplete genes predicted from these contigs. For the 25 PCR-amplified libraries for which an unamplified reference was available, this second tab also includes estimates of assembly errors for each assembly pipeline obtained with QUAST. For the 82 non-viral libraries, the second tab includes the estimated number of bacterial/archaeal genomes and results from genome binning.

10.7717/peerj.6902/supp-7 Table S3 Samples including both unamplified and PCR-amplified libraries List of the 25 PCR-amplified for which an unamplified dataset was available, alongside specific metrics that could be calculated using the unamplified dataset as reference, i.e., correlation of average depth of coverage of contigs, and percentage of contigs from the unamplified assembly detected in the PCR-amplified library. A contig was considered as detected if ≥ 1 read(s) from the PCR-amplified library mapped to it.

## Additional Information and Declarations

### DNA Deposition

The following information was supplied regarding the deposition of DNA sequences: Data are available through the JGI genome portal (https://genome.jgi.doe.gov/portal/). Accession numbers are listed in Table S1.

### Data Availability

The following information was supplied regarding data availability: Reads for the different metagenomes are available on https://genome.jgi.doe.gov/portal/ and the SRA database (https://www.ncbi.nlm.nih.gov/sra), using the links listed in Table S1. Custom perl scripts used in this study are available at https://bitbucket.org/srouxjgi/scripts_pcrlibs_assembly_optimization/src/master/. Results from the different assembly pipelines are available for each library at http://portal.nersc.gov/dna/microbial/prokpubs/BenchmarksPCRMetagenomes/.

## References

[ref-1] Anantharaman K, Breier JA, Dick GJ (2016). Metagenomic resolution of microbial functions in deep-sea hydrothermal plumes across the Eastern Lau Spreading Center. ISME Journal.

[ref-2] Bowers RM, Clum A, Tice H, Lim J, Singh K, Ciobanu D, Ngan CY, Cheng JF, Tringe SG, Woyke T (2015). Impact of library preparation protocols and template quantity on the metagenomic reconstruction of a mock microbial community. BMC Genomics.

[ref-3] Bowers RM, Kyrpides NC, Stepanauskas R, Harmon-Smith M, Doud D, Reddy TBK, Schulz F, Jarett J, Rivers AR, Eloe-Fadrosh EA, Tringe SG, Ivanova NN, Copeland A, Clum A, Becraft ED, Malmstrom RR, Birren B, Podar M, Bork P, Weinstock GM, Garrity GM, Dodsworth JA, Yooseph S, Sutton G, Glöckner FO, Gilbert JA, Nelson WC, Hallam SJ, Jungbluth SP, Ettema TJG, Tighe S, Konstantinidis KT, Liu W-T, Baker BJ, Rattei T, Eisen JA, Hedlund B, McMahon KD, Fierer N, Knight R, Finn R, Cochrane G, Karsch-Mizrachi I, Tyson GW, Rinke C, Lapidus A, Meyer F, Yilmaz P, Parks DH, Eren AM, Schriml L, Banfield JF, Hugenholtz P, Woyke T, Genome Standards Consortium (2017). Minimum information about a single amplified genome (MISAG) and a metagenome-assembled genome (MIMAG) of bacteria and archaea. Nature Biotechnology.

[ref-4] Burstein D, Sun CL, Brown CT, Sharon I, Anantharaman K, Probst AJ, Thomas BC, Banfield JF (2016). Major bacterial lineages are essentially devoid of CRISPR-Cas viral defence systems. Nature Communications.

[ref-5] Campbell BJ, Yu L, Heidelberg JF, Kirchman DL (2011). Activity of abundant and rare bacteria in a coastal ocean. Proceedings of the National Academy of Sciences.

[ref-6] Couradeau E, Sasse J, Goudeau D, Nath N, Hazen TC, Bowen BP, Malmstrom RR, Northen TR (2018). Study of Oak Ridge soils using BONCAT-FACS-Seq reveals that a large fraction of the soil microbiome is active. bioRxiv.

[ref-7] Duhaime MB, Deng L, Poulos BT, Sullivan MB (2012). Towards quantitative metagenomics of wild viruses and other ultra-low concentration DNA samples: a rigorous assessment and optimization of the linker amplification method. Environmental Microbiology.

[ref-8] Duhaime MB, Sullivan MB (2012). Ocean viruses: rigorously evaluating the metagenomic sample-to-sequence pipeline. Virology.

[ref-9] Dutilh BE, Cassman N, McNair K, Sanchez SE, Silva GGZ, Boling L, Barr JJ, Speth DR, Seguritan V, Aziz RK, Felts B, Dinsdale EA, Mokili JL, Edwards RA (2014). A highly abundant bacteriophage discovered in the unknown sequences of human faecal metagenomes. Nature Communications.

[ref-10] Edwards RA, Rohwer F (2005). Viral metagenomics. Nature Reviews. Microbiology.

[ref-11] Eren AM, Esen ÖC, Quince C, Vineis JH, Morrison HG, Sogin ML, Delmont TO (2015). Anvi’o: an advanced analysis and visualization platform for ‘omics data. PeerJ.

[ref-12] Falkowski PG, Fenchel T, Delong EF (2008). The microbial engines that drive earth’s biogeochemical cycles. Science.

[ref-13] Hatzenpichler R, Connon SA, Goudeau D, Malmstrom RR, Woyke T, Orphan VJ (2016). Visualizing in situ translational activity for identifying and sorting slow-growing archaeal-bacterial consortia. Proceedings of the National Academy of Sciences of the United States of America.

[ref-14] Hug LA, Baker BJ, Anantharaman K, Brown CT, Probst AJ, Castelle CJ, Butterfield CN, Hernsdorf AW, Amano Y, Ise K, Suzuki Y, Dudek N, Relman DA, Finstad KM, Amundson R, Thomas BC, Banfield JF (2016). A new view of the tree of life. Nature Microbiology.

[ref-15] Hyatt D, Chen G, Locascio PF, Land ML, Larimer FW, Hauser LJ (2010). Prodigal: prokaryotic gene recognition and translation initiation site identification. BMC Bioinformatics.

[ref-16] John SG, Mendez CB, Deng L, Poulos B, Kauffman AKM, Kern S, Brum J, Polz MF, Boyle EA, Sullivan MB (2011). A simple and efficient method for concentration of ocean viruses by chemical flocculation. Environmental Microbiology Reports.

[ref-17] Kang D, Li F, Kirton ES, Thomas A, Egan RS, An H, Wang Z (2019). MetaBAT 2: an adaptive binning algorithm for robust and efficient genome reconstruction from metagenome assemblies. PeerJ Preprints.

[ref-18] Knowlton C, Veerapaneni R, D’Elia T, Rogers S (2013). Microbial analyses of ancient ice core sections from greenland and antarctica. Biology.

[ref-19] Li H (2015). BFC: correcting illumina sequencing errors. Bioinformatics.

[ref-20] Lloyd KG, Steen AD, Ladau J, Yin J, Crosby L (2018). Phylogenetically novel uncultured microbial cells dominate earth microbiomes. mSystems.

[ref-21] Marine R, McCarren C, Vorrasane V, Nasko D, Crowgey E, Polson SW, Wommack KE (2014). Caught in the middle with multiple displacement amplification: the myth of pooling for avoiding multiple displacement amplification bias in a metagenome. Microbiome.

[ref-22] Mikheenko A, Prjibelski A, Saveliev V, Antipov D, Gurevich A (2018). Versatile genome assembly evaluation with QUAST-LG. Bioinformatics.

[ref-23] Neuwirth E (2014). RColorBrewer: ColorBrewer Palettes.

[ref-24] Nurk S, Bankevich A, Antipov D, Gurevich AA, Korobeynikov A, Lapidus A, Prjibelski AD, Pyshkin A, Sirotkin A, Sirotkin Y, Stepanauskas R, Clingenpeel SR, Woyke T, McLean JS, Lasken R, Tesler G, Alekseyev MA, Pevzner PA (2013). Assembling single-cell genomes and mini-metagenomes from chimeric MDA products. Journal of Computational Biology.

[ref-25] Nurk S, Meleshko D, Korobeynikov A, Pevzner PA (2017). metaSPAdes: a new versatile metagenomic assembler. Genome Research.

[ref-26] Parks DH, Imelfort M, Skennerton CT, Hugenholtz P, Tyson GW (2015). CheckM: assessing the quality of microbial genomes recovered from isolates, single cells, and metagenomes. Genome Research.

[ref-27] Parks DH, Rinke C, Chuvochina M, Chaumeil P, Woodcroft BJ, Evans PN, Hugenholtz P, Tyson GW (2017). Recovery of nearly 8,000 metagenome-assembled genomes substantially expands the tree of life. Nature Microbiology.

[ref-28] R Core Team (2018). https://www.r-project.org.

[ref-29] Raes J, Bork P (2008). Molecular eco-systems biology: towards an understanding of community function. Nature Reviews. Microbiology.

[ref-30] Reyes GR, Kim JP (1991). Sequence-independent, single-primer amplification (SISPA) of complex DNA populations. Molecular and Cellular Probes.

[ref-31] Rinke C, Low S, Woodcroft BJ, Raina J-B, Skarshewski A, Le XH, Butler MK, Stocker R, Seymour J, Tyson GW, Hugenholtz P (2016). Validation of picogram- and femtogram-input DNA libraries for microscale metagenomics. PeerJ.

[ref-32] Rinke C, Schwientek P, Sczyrba A, Ivanova NN, Anderson IJ, Cheng JF, Darling A, Malfatti S, Swan BK, Gies EA, Dodsworth JA, Hedlund BP, Tsiamis G, Sievert SM, Liu W-T, Eisen JA, Hallam SJ, Kyrpides NC, Stepanauskas R, Rubin EM, Hugenholtz P, Woyke T (2013). Insights into the phylogeny and coding potential of microbial dark matter. Nature.

[ref-33] Roux S, Brum JR, Dutilh BE, Sunagawa S, Duhaime MB, Loy A, Poulos BT, Solonenko N, Lara E, Poulain J, Pesant S, Kandels-Lewis S, Dimier C, Picheral M, Searson S, Cruaud C, Alberti A, Duarte CM, Gasol JM, Vaqué D, Bork P, Acinas SG, Wincker P, Sullivan MB, Tara Oceans Coordinators (2016a). Ecogenomics and potential biogeochemical impacts of uncultivated globally abundant ocean viruses. Nature.

[ref-34] Roux S, Solonenko NE, Dang VT, Poulos BT, Schwenck SM, Goldsmith DB, Coleman ML, Breitbart M, Sullivan MB (2016b). Towards quantitative viromics for both double-stranded and single-stranded DNA viruses. PeerJ.

[ref-35] Schloissnig S, Arumugam M, Sunagawa S, Mitreva M, Tap J, Zhu A, Waller A, Mende DR, Kultima JR, Martin J, Kota K, Sunyaev SR, Weinstock GM, Bork P (2013). Genomic variation landscape of the human gut microbiome. Nature.

[ref-36] Schloss PD, Girard RA, Martin T, Edwards J, Thrash JC (2016). Status of the archaeal and bacterial census: an update. MBio.

[ref-37] Solonenko SA, Ignacio-Espinoza JC, Alberti A, Cruaud C, Hallam S, Konstantinidis K, Tyson G, Wincker P, Sullivan MB (2013). Sequencing platform and library preparation choices impact viral metagenomes. BMC Genomics.

[ref-38] Spang A, Saw JH, Jørgensen SL, Zaremba-Niedzwiedzka K, Martijn J, Lind AE, Van Eijk R, Schleper C, Guy L, Ettema TJG (2015). Complex archaea that bridge the gap between prokaryotes and eukaryotes. Nature.

[ref-39] Steward GF, Culley AI, Wilhelm SW, Weinbauer MG, Suttle CA (2010). Extraction and purification of nucleic acids from viruses. Manual of aquatic viral ecology American society of limnology and oceanography.

[ref-40] Sunagawa S, Coelho LP, Chaffron S, Kultima JR, Labadie K, Salazar G, Djahanschiri B, Zeller G, Mende DR, Alberti A, Cornejo-Castillo FM, Costea PI, Cruaud C, d’Ovidio F, Engelen S, Ferrera I, Gasol JM, Guidi L, Hildebrand F, Kokoszka F, Lepoivre C, Lima-Mendez G, Poulain J, Poulos BT, Royo-Llonch M, Sarmento H, Vieira-Silva S, Dimier C, Picheral M, Searson S, Kandels-Lewis S, Bowler C, de Vargas C, Gorsky G, Grimsley N, Hingamp P, Iudicone D, Jaillon O, Not F, Ogata H, Pesant S, Speich S, Stemmann L, Sullivan MB, Weissenbach J, Wincker P, Karsenti E, Raes J, Acinas SG, Bork P, Tara Oceans coordinators (2015). Ocean plankton. Structure and function of the global ocean microbiome. Science.

[ref-41] Suttle CA (2007). Marine viruses–major players in the global ecosystem. Nature Reviews. Microbiology.

[ref-42] The Uniprot Consortium D (2019). UniProt: a worldwide hub of protein knowledge. Nucleic Acids Research.

[ref-43] Torchiano M (2017). effsize: efficient effect size computation.

[ref-44] Trubl G, Solonenko N, Chittick L, Solonenko SA, Rich VI, Sullivan MB (2016). Optimization of viral resuspension methods for carbon-rich soils along a permafrost thaw gradient. PeerJ.

[ref-45] Tyson GW, Chapman J, Hugenholtz P, Allen EE, Ram RJ, Richardson PM, Solovyev VV, Rubin EM, Rokhsar DS, Banfield JF (2004). Community structure and metabolism through reconstruction of microbial genomes from the environment. Nature.

[ref-46] Weinmaier T, Probst AJ, La Duc MT, Ciobanu D, Cheng JF, Ivanova N, Rattei T, Vaishampayan P (2015). A viability-linked metagenomic analysis of cleanroom environments: eukarya, prokaryotes, and viruses. Microbiome.

[ref-47] Wickham H (2016). https://cran.r-project.org/web/packages/dplyr/index.html.

[ref-48] Wickham H, François R, Henry L, Müller K (2018).

[ref-49] Yokouchi H, Fukuoka Y, Mukoyama D, Calugay R, Takeyama H, Matsunaga T (2006). Whole-metagenome amplification of a microbial community associated with scleractinian coral by multiple displacement amplification using *φ*29 polymerase. Environmental Microbiology.

