# Supplementary figures and images for: Optimizing de novo genome assembly from PCR-amplified metagenomes
Source: PeerJ. 2019 May 9;7:e6902. doi: 10.7717/peerj.6902 (PMC6511391; doi:10.7717/peerj.6902)

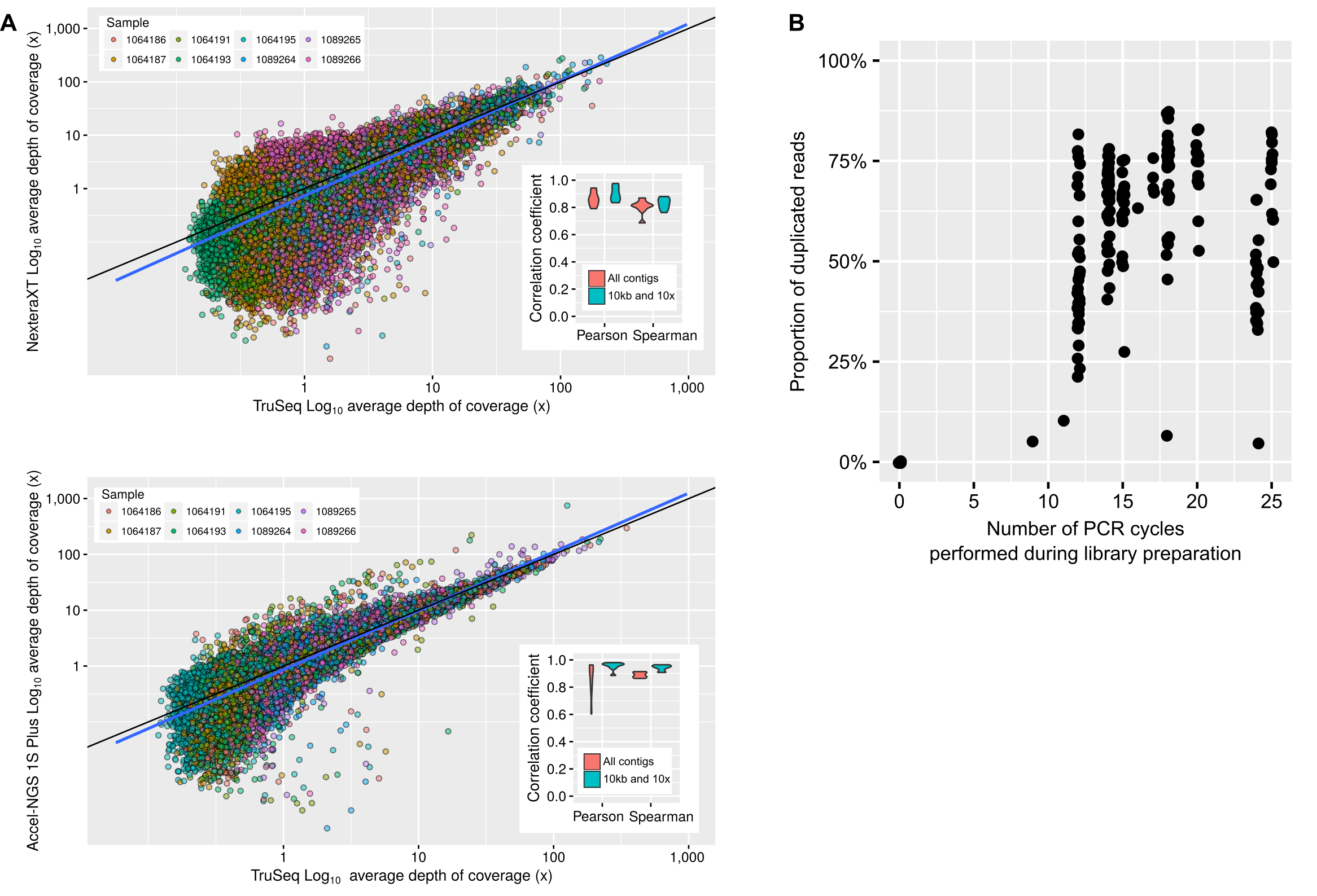

Supplement: Figure S1 — (A) Comparison of depth of coverage between unamplified (TruSeq, x-axis) and PCR-amplified (Nextera XT or Accel-NGS 1S Plus, y-axis) libraries. The average depth of coverage was computed for each contig as the average read depth normalized by the total size of the library. The 1:1 equivalence is indicated with a black line, while a linear best fit is shown in blue. For clarity, only 1,000 contigs randomly selected from each sample are plotted. Contigs with no reads mapped in the PCR-amplified library were not included. To be able to directly compare the two plots, only samples for which both a Nextera XT and 1S Plus libraries were available are included (Table S1). The subpanels show the correlation coefficient (Pearson and Spearman) of a sample-by-sample correlation between depth of coverage in unamplified and PCR-amplified libraries, either for all contigs or only for contigs ≥ 10 kb with a depth of coverage ≥10 ×. (B) Percentage of duplicated reads (y-axis) as a function of the number of PCR cycles performed during library creation (x-axis). Underlying data are availabe in Table S1. [file peerj-07-6902-s001.png]

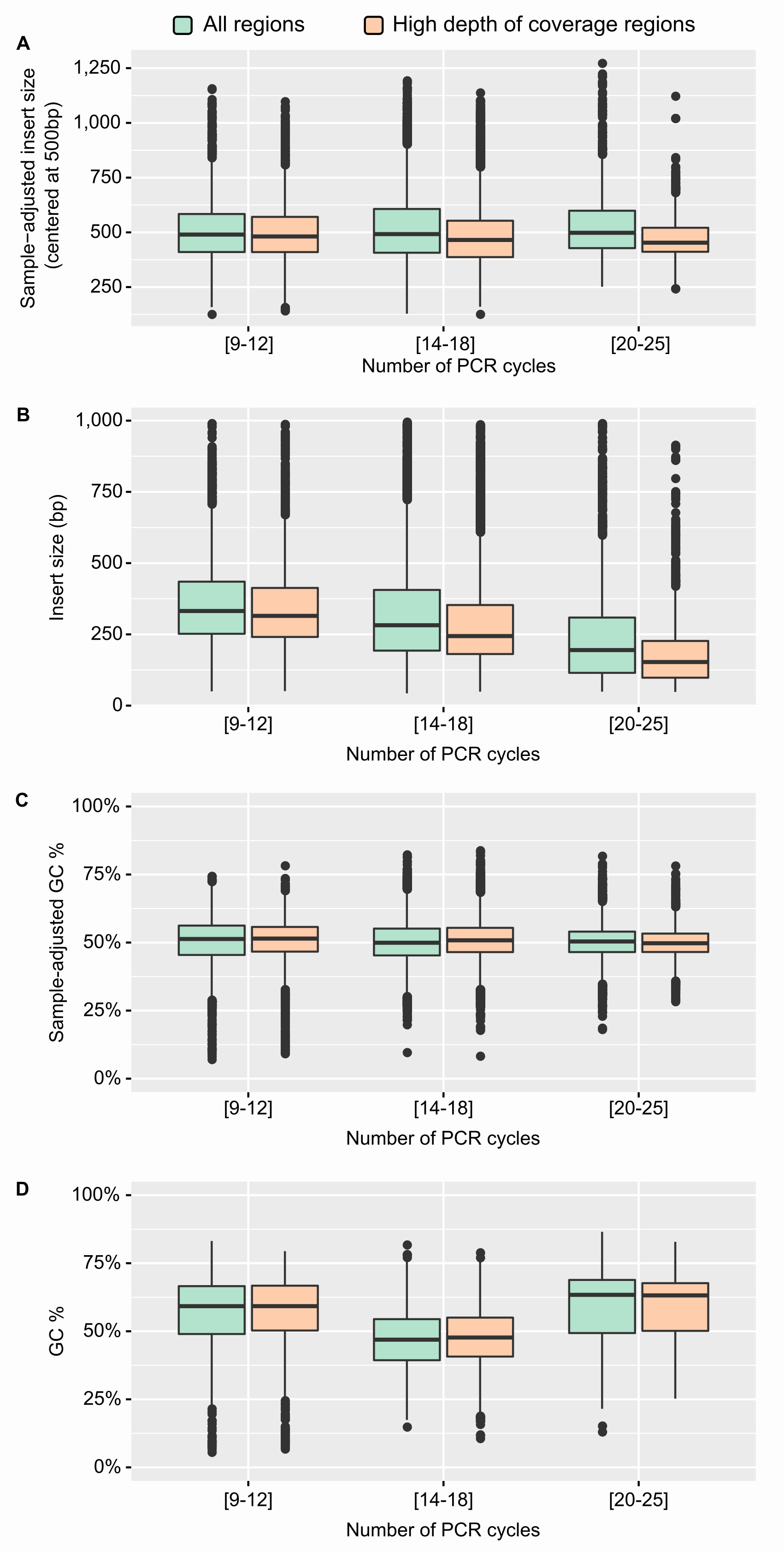

Supplement: Figure S2 — (A & B) Distribution of insert size for all regions (green) or only regions with high depth of coverage (orange) across PCR-amplified libraries. In panel A, all insert sizes were centered around 500 bp to enable a more direct comparison between libraries. Panel B shows the same data without this transformation (i.e. raw insert size). (C & D) Distribution of GC % for all regions (green) or only regions with high depth of coverage (orange). For panel C, each library GC% was centered around 50%, while panel D shows the same data without this transformation. [file peerj-07-6902-s002.png]

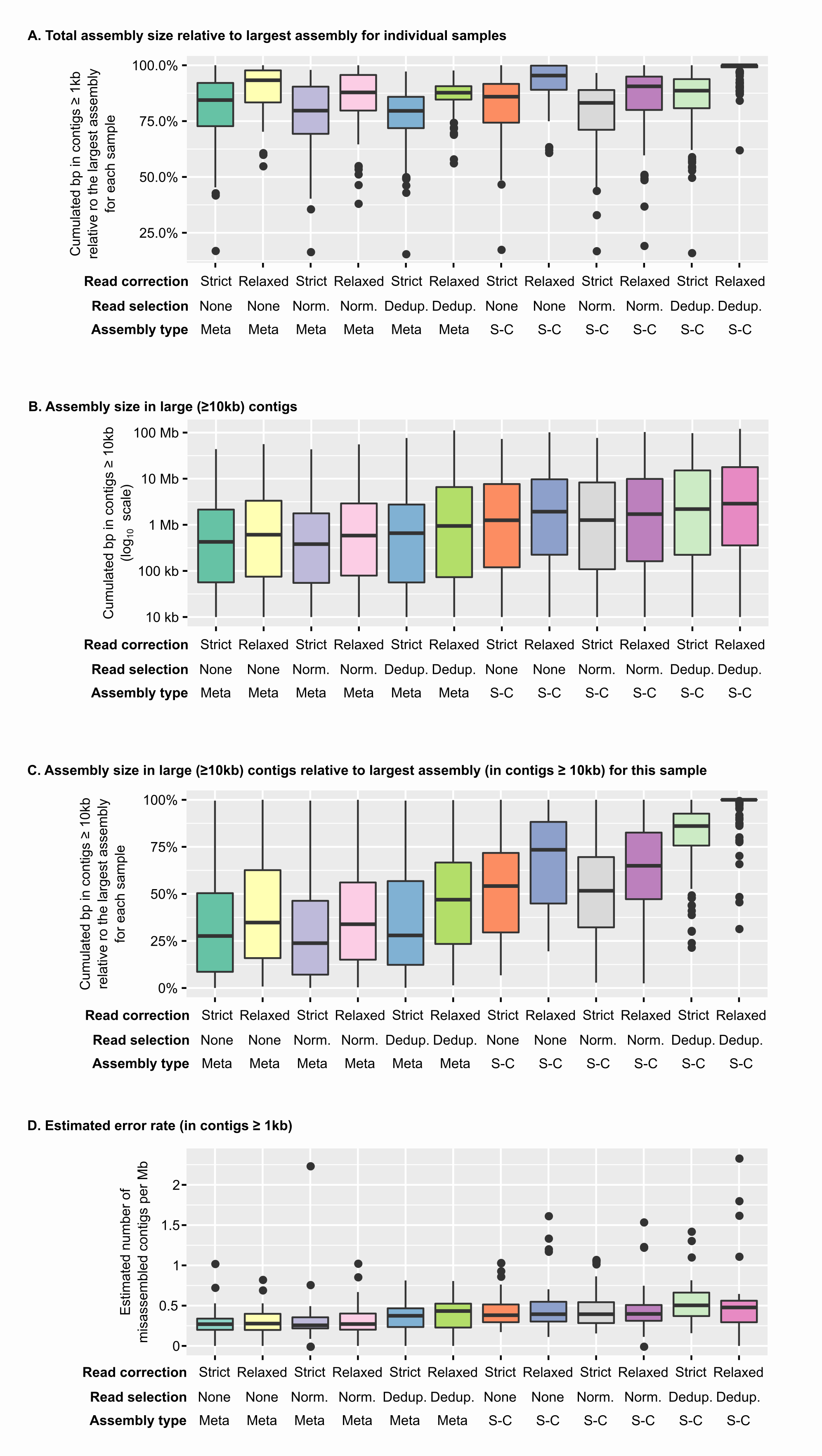

Supplement: Figure S3 — Comparisonof the output of different assembly pipelines applied to PCR-amplified libraries. Panels A & B show the cumulative length of all contigs (A) or contigs ≥ 10 kb (B) across assembly pipelines (x-axis). Panel C displays the cumulative length of contigs ≥ 10 kb relative to the largest value for each library, i.e. as a percentage of the “best” assembly for this library (“best” being defined as the largest cumulative length of contigs ≥ 10 kb). Panel D displays the distribution of estimated error rates across the different assembly pipelines, for the 25 libraries for which error rates could be estimated (Tables S2 & S3). Underlying data for individual assemblies are available in Table S2. Norm.: Normalization, Dedup.: Deduplication, Meta: metaSPAdes, SC: single-cell SPAdes. [file peerj-07-6902-s003.png]

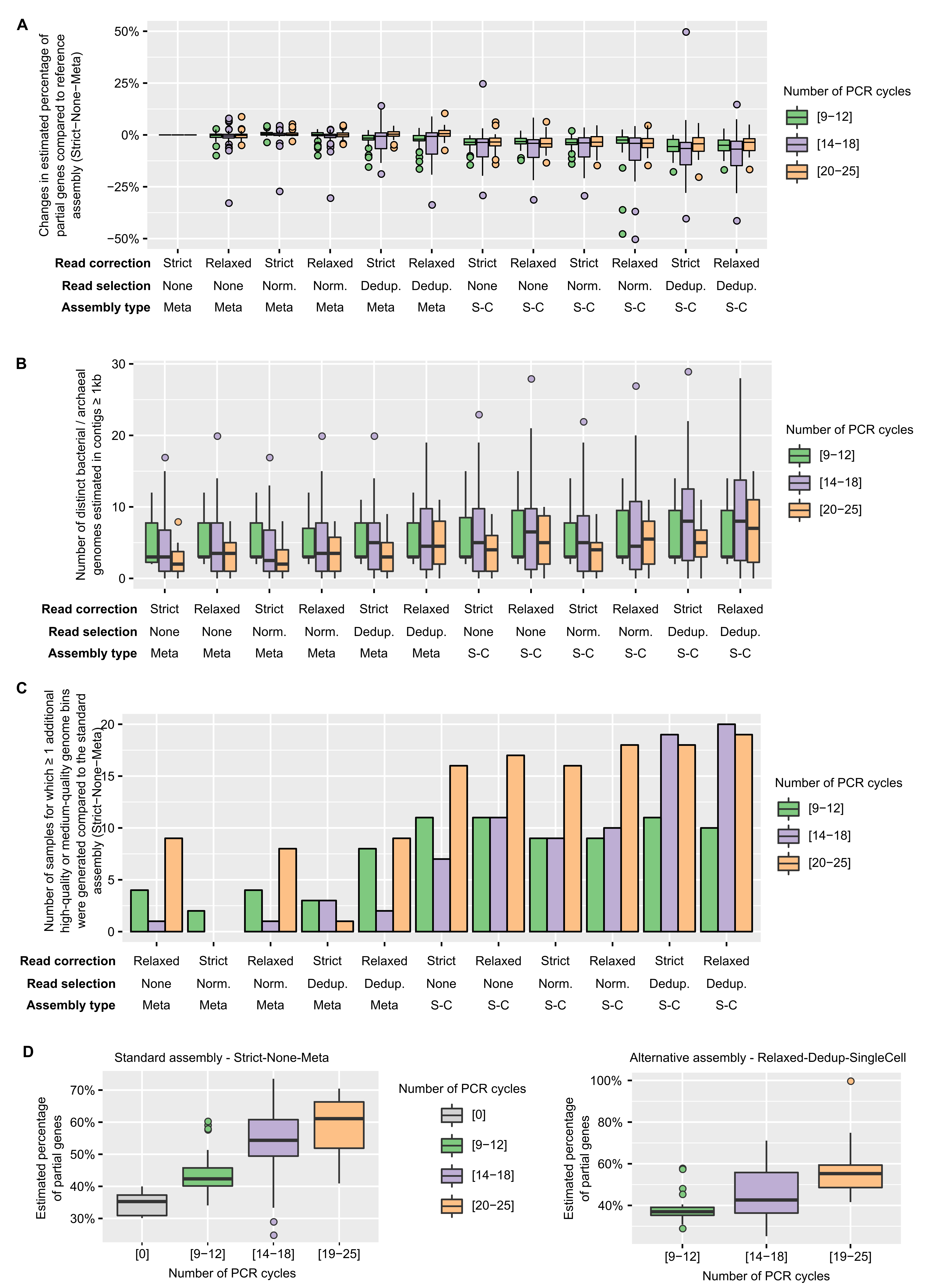

Supplement: Figure S4 — (A) Link between assembly methods choice and ratio of incomplete gene. The boxplot displays the estimated ratio of incomplete genes (see Methods) in each assembly relative to this ratio in the “standard” metagenome assembly (Strict-None-Meta). (B) Estimated number of bacterial and archaeal genomes in assemblies based on known single-copy marker genes. (C) Number of samples for which ≥ 1 high-quality or medium-quality genome bin(s) (bacterial or archaeal) was obtained. (D) Comparison of the ratio of incomplete genes for different number of PCR cycles, for contigs obtained using the “standard” assembly pipeline (“Strict-None-Meta, ”left panel) or the proposed “optimized” pipeline (“Relaxed-Dedup-SingleCell”, right panel). Underlying data for individual assemblies are available in Table S2. Norm.: Normalization, Dedup.: Deduplication, Meta: metaSPAdes, SC: single- cell SPAdes. [file peerj-07-6902-s004.png]
